# Supplementary material for: The occurrence of Aerococcus urinaeequi and non-aureus staphylococci in raw milk negatively correlates with Escherichia coli clinical mastitis
Source: mSystems. 2024 Sep 10;9(10):e00362-24. doi: 10.1128/msystems.00362-24 (PMC11494980; doi:10.1128/msystems.00362-24)
Supplement: Supplemental Figures — Figures S1 to S6. [file msystems.00362-24-s0001.docx]

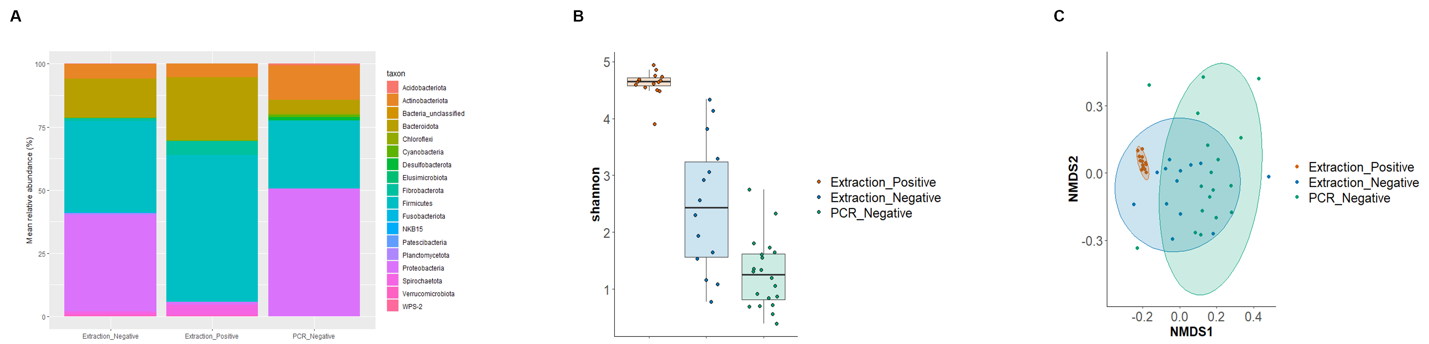


**Fig S1. Relative abundance and diversity of the microbiome from the negative and positive controls, and PCR control.** (A) The differences in the relative abundance of taxa were averaged between all positive and negative controls and shown as a stacked bar graph. The Shannon index is plotted as a measure of alpha-diversity (B) and an NMDS plot is used to visualize differences in beta-diversity (C) for each positive and negative control sample.


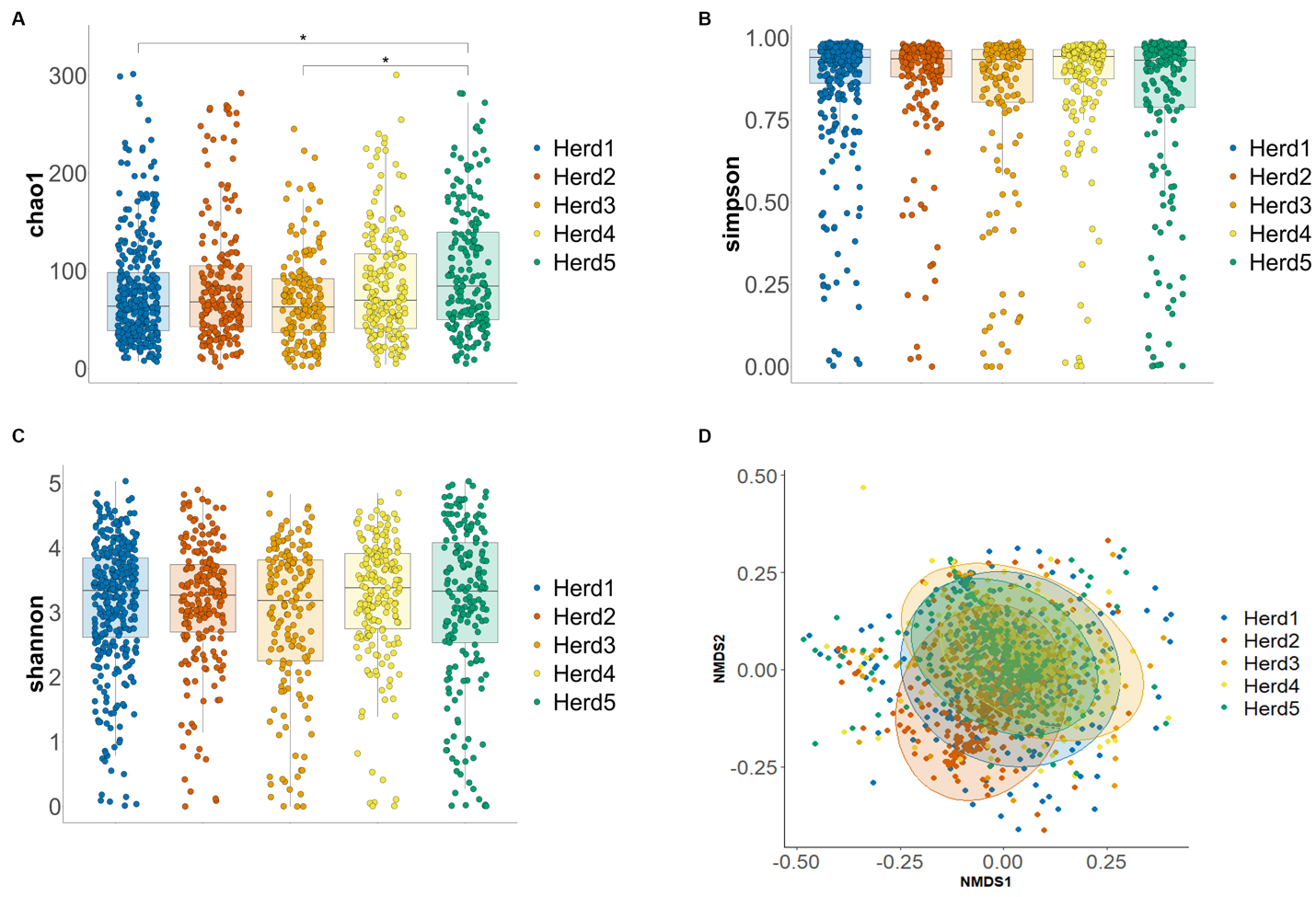


**Fig S2. Alpha- and beta-diversities of raw milk microbiome at herd-level.** Differences in alpha-diversity are visualized between herds by plotting the Chao 1 (A), Simpson (B), and Shannon (C) indexes for each raw milk sample. (D) Beta-diversity is visualized using an NMDS plot with eclipses shown containing samples from each individual herd.


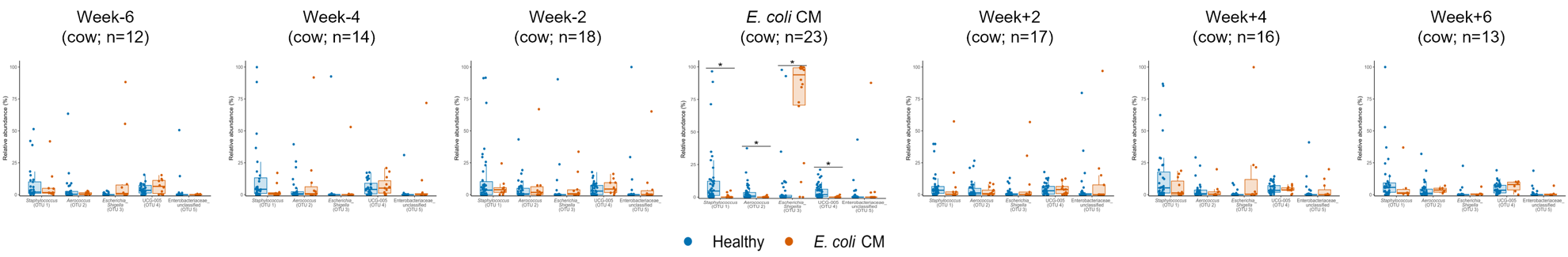


**Fig S3. Longitudinal differential abundance for the five most common OTUs.** The relative abundance is shown for OTU0001 (*Staphylococcus*), OTU0002 (*Aerococcus*), OTU0003 (*Escherichia*_*Shigella*),­­ OTU0004 (UCG_005), and OTU0005 (unclassified *Enterobacteriaceae*) to allow comparison between health milk samples and milk samples taken from cattle with active *E. coli* mastitis.


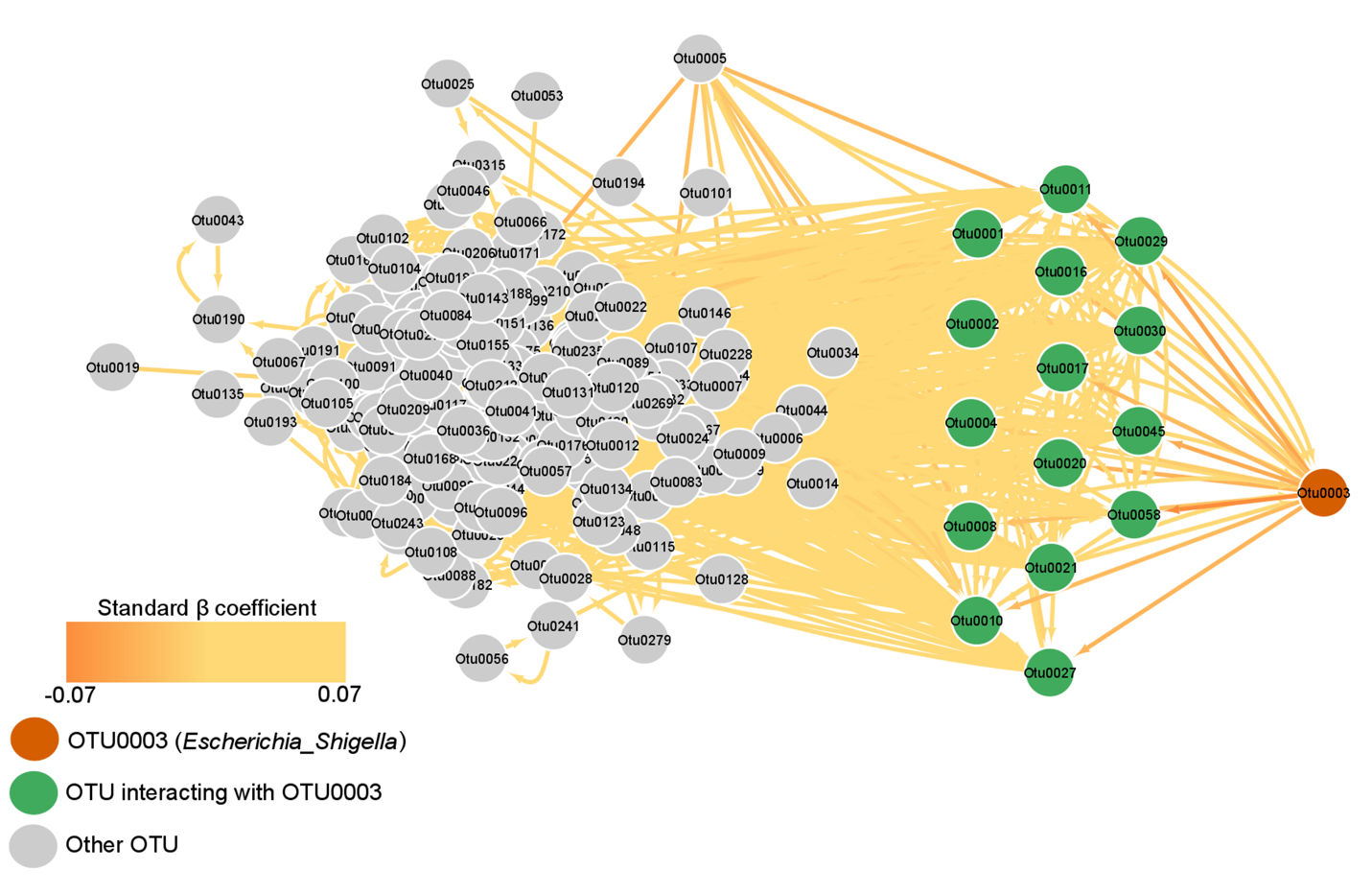


**Fig S4. Microbial network of 186 OTUs in the raw milk samples.** Each arrow indicates the direction of the relationship between OTUs based on β calculation and GLM, and the colour of the arrow indicates the strength of either positive or negative relationships based on standard β-coefficient values. In total, 1,795 interactions were detected and 23 of them were interactions between 16 OTUs and OTU0003 (*Escherichia_Shigella*). The phylogenetic identity at the genus level, of each of the OTUs identified in this figure are listed in Table S5.

**
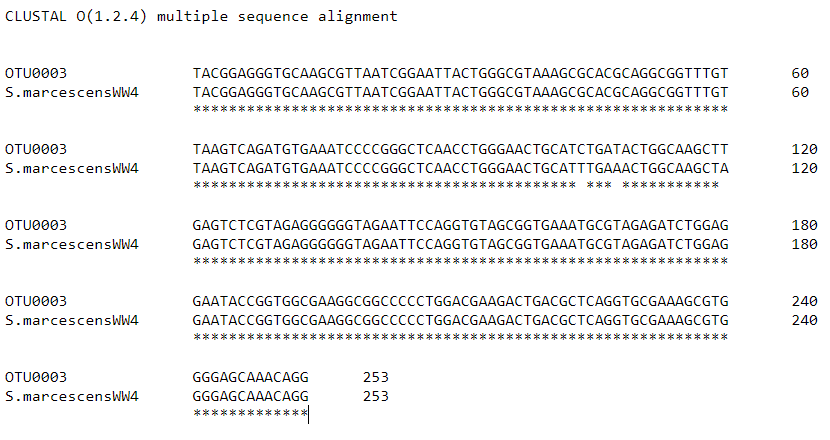
**

**Fig S5. Sequence alignment of the V4 region of 16S rRNA gene in OTU0003 and *S. marcescens* WW4.** The sequence alignment indicates that our 16s rRNA TAS results would not be able to differentiate *E. coli* and *S. marcescens.*

**
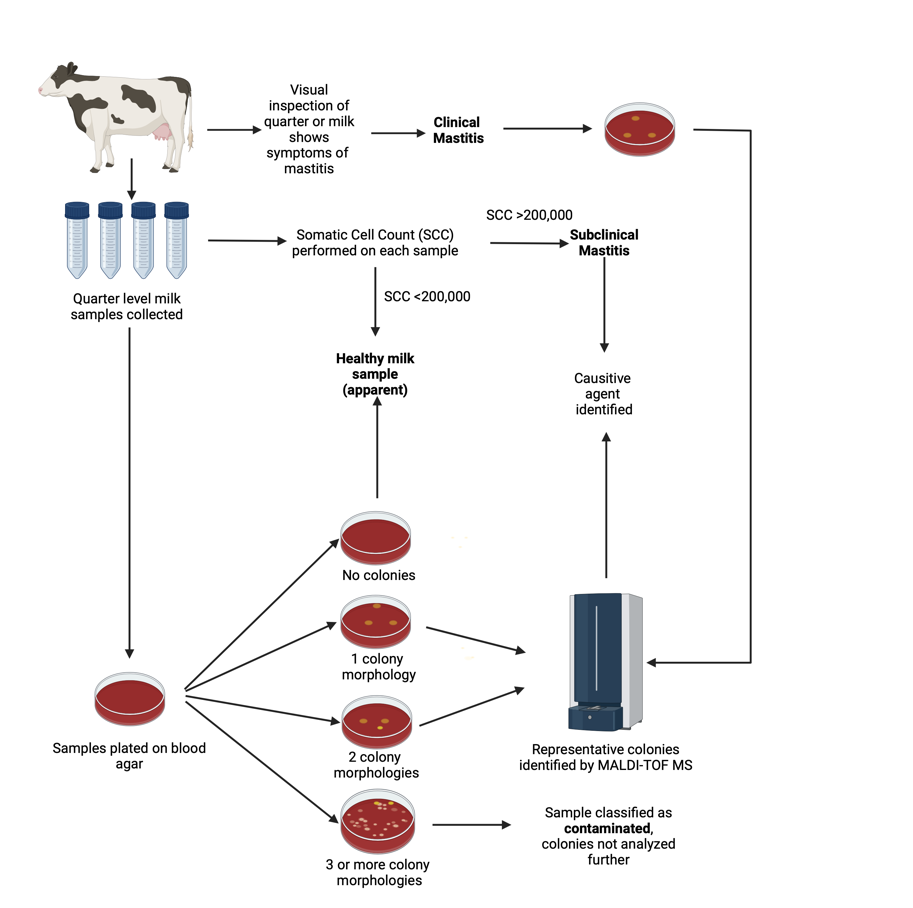
**

**Fig S6. Sample classification decision chart.** Samples milk samples were classified as being from a quarter with CM if they were taken from a quarter with symptoms of CM. Milk samples taken from quarters with symptoms were cultured on blood agar and the colonies were identified using MALDI-TOF. Samples taken from putatively healthy quarters were cultured on blood agar. For samples which produced 2 or less distinct colony morphologies, each colony was identified using MALDI-TOF. Samples which produced greater than 2 distinct morphologies were classified as contaminated, and colonies were not identified.
